# De novo transcriptome sequencing and gene expression profiling of spinach (*Spinacia oleracea* L.) leaves under heat stress

**DOI:** 10.1038/srep19473

**Published:** 2016-02-09

**Authors:** Jun Yan, Li Yu, Jiping Xuan, Ying Lu, Shijun Lu, Weimin Zhu

**Affiliations:** 1 Horticulture Research Institute, Shanghai Academy Agricultural Sciences; Key Laboratory of Protected Horticulture Technology, Shanghai, 201403, China; 2 Institute of Botany, Jiangsu Province and the Chinese Academy of Sciences, Nanjing, Jiangsu Province, 210014, China

## Abstract

Spinach (*Spinacia oleracea*) has cold tolerant but heat sensitive characteristics. The spinach variety ‘Island,’ is suitable for summer periods. There is lack molecular information available for spinach in response to heat stress. In this study, high throughput de novo transcriptome sequencing and gene expression analyses were carried out at different spinach variety ‘Island’ leaves (grown at 24 °C (control), exposed to 35 °C for 30 min (S1), and 5 h (S2)). A total of 133,200,898 clean reads were assembled into 59,413 unigenes (average size 1259.55 bp). 33,573 unigenes could match to public databases. The DEG of controls vs S1 was 986, the DEG of control vs S2 was 1741 and the DEG of S1 vs S2 was 1587. Gene Ontology (GO) and pathway enrichment analysis indicated that a great deal of heat-responsive genes and other stress-responsive genes were identified in these DEGs, suggesting that the heat stress may have induced an extensive abiotic stress effect. Comparative transcriptome analysis found 896 unique genes in spinach heat response transcript. The expression patterns of 13 selected genes were verified by RT-qPCR (quantitative real-time PCR). Our study found a series of candidate genes and pathways that may be related to heat resistance in spinach.

Agricultural production is threatened by abiotic stresses such as drought, extreme temperatures, flooding and chemical toxicity^1^. Heat stress can reduce crop production, and studies suggest that these reductions will become a major issue in coming years due to global warming^2^. Global warming predictions foretell that temperatures will rise further by 2–6 °C by the end of this century due to the rapid increase in atmospheric greenhouse gas concentrations^3^. Many studies reveal that heat resistance depends on species and genotype. However, there were some differences in response to heat stress among different species or genotype^4^. Therefore, it is essential to reveal molecular mechanism of crop plants in response to heat stress, which is helpful to breed heat-tolerant crop plants^4^.

Next-generation sequencing (NGS) technologies, such as Illumina/Solexa (San Diego, CA, USA), the SOLiD platform from Applied Biosystems, and Roche 454 sequencing, have revolutionized genomics because they allow faster and less expensive sequencing^5^. NGS-based RNA-Seq methods, which are used for transcriptome analysis, even allow simultaneous acquisition of sequences^6^. These fast sequencing methods are used to characterize genes, detect gene expression pattern and level, recognize and quantify rare transcripts without prior information of the particular gene or reference genome, and generate information on alternative splicing and sequence variations in identified genes^6^,^7^. In recent years, RNA-Seq has been successfully used to reveal the complex biotic and abiotic stress response mechanism in many plant species, such as grape (*Vitis vinifera*)^8^, cotton (*Gossypium hirsutum*)^9^, cucumber (*Cucumis sativus*)^10^, *Ammopiptanthus mongolicus*^11^, and *Arabidopsis thaliana*^12^. However, data on the molecular responses to heat stress in spinach leaves are very limited.

Spinach (*Spinacia oleracea*) belongs to the family Amaranthaceae and is an economically important vegetable crop that is grown worldwide. Spinach has cold tolerant but heat sensitive characteristics so that the crop may undergo heat stress throughout its entire life cycle. High temperatures affect spinach plant growth and significantly decrease yield and quality. Recently, some spinach varieties such as ‘Menorca’ and ‘Island,’ that originate from Seminis Vegetable Seeds Inc. have been shown to be suitable for summer periods. In the present study, the genome-wide analysis of gene expression during heat treatment was performed on the ‘Island’ summer spinach variety via a NGS-based Illumina paired-end sequencing platform. This study will aid in understanding the heat stress response in spinach at the molecular level, and enriches the genomic information for spinach.

## Results and Discussion

### Illumina sequencing and de novo assembly

**Figure 1:**
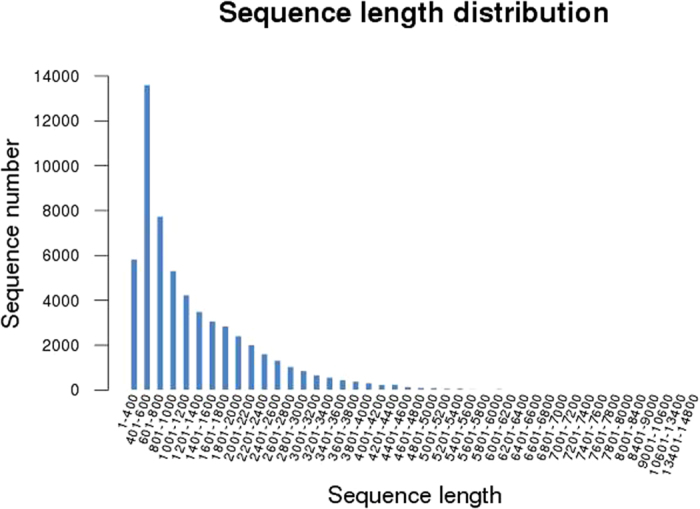
Length distribution of the assembled transcripts.

Three cDNA libraries were generated with mRNA from three samples: control (grown at 24 °C), S1 (exposed at 35 °C for 30 min) and S2 (exposed at 35 °C for 5 h) spinach leaf. These cDNA libraries were then subjected to Illumina deep-sequencing. The raw reads were deposited in the Sequence Read Archive at GenBank databases ID: SRP051935. A total of 44,682,577, 42,290,681, and 46,227,640 clean reads with 4,349,881,212, 4,122,064,627, and 4,508,391,358 nucleotides were obtained from the control, S1, and S2, respectively. The clean reads were >12 gigabases (total length), which was equivalent to ~12-fold coverage of the genome of *S. oleracea*. All clean reads were *de novo* assembles with the Trinity method^13^ because *S. oleracea* does not have an appropriate reference genome sequence. Table 1 shows more than 80% of the reads could be mapped back to the assembled transcripts. Finally, a total of 133,200,898 clean reads were assembled into 59,413 unigenes with a total unigene length of 74,833,448 bp. The length of the unigenes ranged from 351–14,797 bp, with an average unigene size of 1259.55 bp. There were 26,684 unigenes (44.91%) in the length range of 401–1000 bp, 23,655 unigenes (39.81%) in the length range of 1001–3200 bp, and 3,233 unigenes (5.44%) with length >3200 bp (Fig. 1). The length distribution of Arabidopsis and rice (*Oryza sativa*) transcriptome sequences were obtained from publicly available datasets. Comparison analysis indicated that the length range of most unigenes was 400–3200 bp in these three species (Table 2).

ORF prediction analysis was made using get ORF from EMBOSS package. There were 35,805 (60.26%) unigenes were identified to have an ORF. These unigenes were compared with 31,282 sugar beet (*Beta vulgaris*) transcriptome sequences obtained from publicly available datasets which have an ORF (E-value <1e^−10^). The result show that 12,307 unigenes had significant matched to sugar beet transcripts, and there were 23,498 specific sequence in spinach compared to sugar beet transcripts.

**Figure 2:**
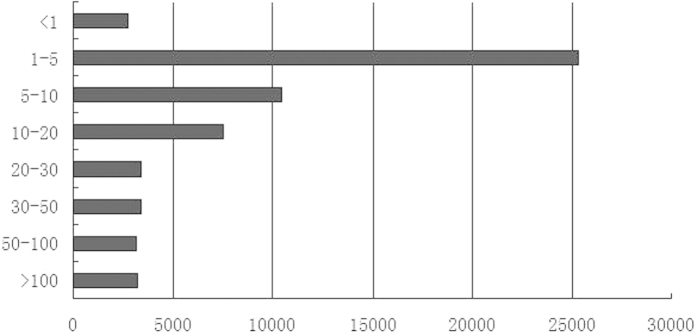
A histogram of the average read-depth coverage for unigenes. The x-axis indicates the number of unigenes, and the y-axis indicates the distribution of read-depth coverage.

The web-based tool ESTcal^14^ was used to assess the breadth and depth of all unigenes because *S. oleracea* does not have transcriptome profile for comparison. The average read-depth coverage was 31.65-fold, and 22.28% unigenes was greater than 20 fold (Fig. 2), which suggested that these unigenes have high contiguity and high coverage. So these unigenes could be used in further analyses.

### Functional annotation and classification of spinach leaf transcriptome

Public databases (NR, NT, SwissProt, and KEGG) was used to annotated all unigenes with local BLAST programs (E value < 1.0E-5). The results showed that 33,573 (56.51% of 59,413) unigenes were matched to one or more of the databases. The large amount of unannotated unigenes generated in this study may be assembled uncorrectly or represent a specific gene pool for spinach leaf studies^6^. These unannotated unigenes may be useful for further study of heat stress response mechanisms and identification of novel genes in *S. oleracea*.

**Figure 3:**
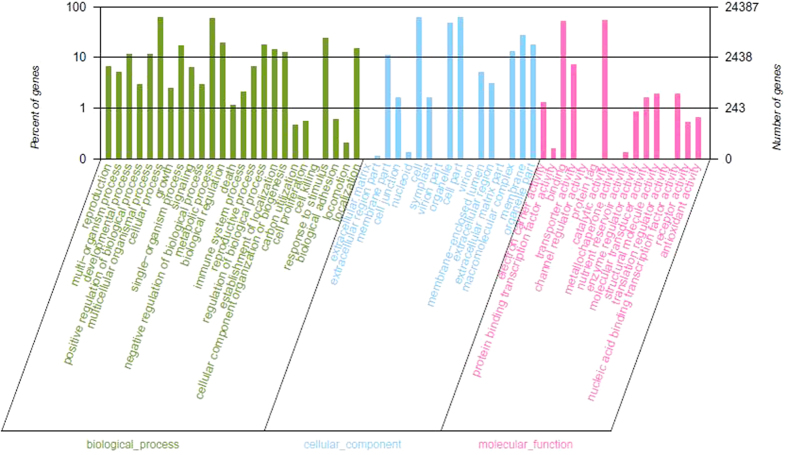
Gene ontology classification of the *S. oleracea* transcriptome.

GO (http://www.geneontology.org/) is an international classification system for standardized gene functions, which have three GO categories: biological process, molecular function, and cellular component. Based on sequence homology, a total of 24,387 (41.05% of 59,413) unigenes were assigned to one or more GO term annotations (Fig. 3). Among which, ‘cell’ (15,020; 61.59% of 24,387), ‘cell part’ (15,020; 61.59% of 24,387), and ‘organelle’ (11,734; 44.12% of 24,387) were the terms that dominated in the cellular component category. ‘Cellular process’ (14,905; 61.11% of 24,387), ‘metabolic process’ (14,592; 59.84% of 24,387), and ‘response to stimulus’ (5731; 23.50% of 24,387) were the most representative terms in the biological process category. ‘Binding’ (12,444, 51.03% of 24,387) and ‘catalytic activity’ (13,374, 58.84% of 24,387) were the most abundant terms in the molecular function category. Only a few unigenes (less than 10) were clustered into the terms of ‘channel regulator activity,’ ‘protein tag,’ ‘metallochaperone activity,’ ‘translation regulator activity,’ ‘virion part,’ ‘extracellular matrix part,’ and ‘cell killing’. The GO terms in this study were compared with sugar beet GO terms which were obtained from publicly available datasets. Most GO terms of spinach were the same as sugar beet. In contrast to sugar beet, four GO terms were specific in spinach including ‘channel regulator activity’, ‘death’, ‘carbon utilization’ and ‘cell proliferation’. We speculate that the specific GO terms of spinach may result from heat stress or species differences.

**Figure 4:**
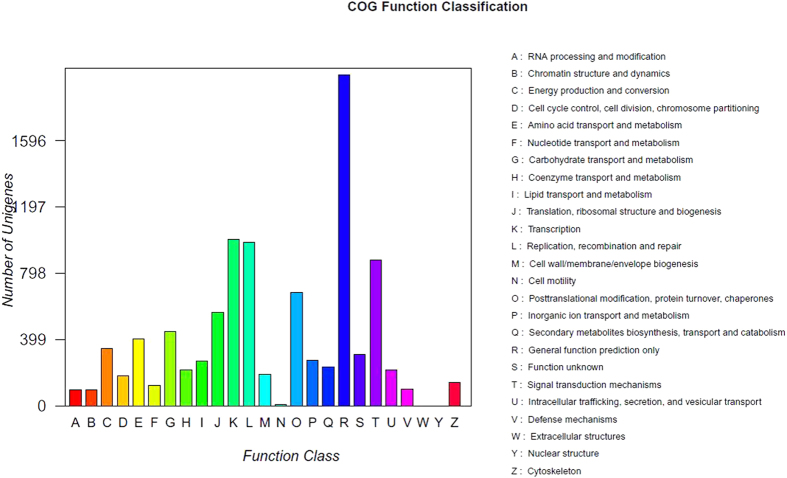
COG functional classification of the *S. oleracea* transcriptome.

All unigenes were match with the Cluster of Orthologus Groups (COG database) for functional prediction and classification. A total of 9,760 (16.43% of 59,413) unigenes were assigned appropriate COG clusters, which could be classified into 25 functional categories (Fig. 4). Among them, the largest category was ‘General function prediction only’ (20.42%); followed by ‘transcription’ (10.26%); ‘Replication, recombination, and repair’ (10.07%); ‘Signal transduction mechanisms’ (9.0%); and ‘Posttranslational modification, protein turnover, and chaperones’ (6.98%).

To identify biological pathways activated in spinach leaves under heat stress, all unigenes were annotated and mapped to the KEGG database (http://genome.jp/kegg/). Out of 33,573 total annotated unigenes, 10,143 unigenes were significantly matched to KEGG database and were assigned to 292 KEGG pathways (see Supplementary Table S1). The major pathways in this study were ‘metabolic pathways [ko01100]’(2,5525.22% of 10,143), ‘biosynthesis of secondary metabolites [ko01110]’(1,088, 10.73%8, of 10,143), ‘microbial metabolism in diverse environments [ko01120]’(439, 4.33% of 10,143), ‘spliceosome [ko03040]’(330, 3.25% of 10,143) and ‘ribosome [ko03010]’(272, 2.68% of 10,143). Furthermore, 975 unigenes were involved in the plant biotic/abiotic defense, which includes ‘MAPK signaling pathway’, ‘phosphatidylinositol’, ‘signaling system’, ‘ascorbate and aldarate metabolism’, ‘calcium signaling pathway’, ‘plant-pathogen interaction’, ‘glutathione’ metabolism’, ‘plant hormone signal transduction’, ‘ABC transporters’, and ‘phenylalanine metabolism’.

### Differentially expressed genes (DEGs) involved in the heat stress response of spinach leaves

In order to found the differentially expressed genes in spinach leaves responding to heat stress among control, S1, and S2, clean reads of these libraries were respectively assigned to all unigenes using the RSEM (RNA-Seq by Expectation Maximization) software^15^, and the difference expression levels of unigenes were calculated with the FPKM method^16^. The DEGs were determined with the absolute change value of |log FC| > 2 using a greater statistical significance level (P < 0.05) and false discovery rates (FDR < 0.001). The DEG of controls vs S1 was 986, the DEG of controls vs S2 was 1741, and the DEG of S1 vs S2 was 1587. The number of up-regulated unigenes in the S1 and S2 samples compared with control was 550 and 1,131, respectively.

### Validation of the differentially expressed genes (DEGs) via RT-qPCR

In order to experimentally confirm that the differentially expressed genes obtained in this study were credible, 13 DEGs from different gene families (including 8 up-regulated and 5 down-regulated in S1 or S2) were selected and their expression pattern was examined via RT-qPCR. The RT-qPCR results show that the expression of all these DEGs was similar to those obtained from the Illumina sequencing analysis (see Supplementary Table S2). Moreover, the fold-changes obtained by DEG were generally greater than those obtained by RT-qPCR, which was a universal phenomenon in other studies and attributed to the essentially different of algorithms and sensitivity between the two techniques^17^,^18^. These results indicated that the method used to determine DEGs in this study were valid.

### GO functional enrichment and KEGG pathway enrichment analysis of DEGs

Goatools (https://github.com/tanghaibao/goatools) was used to identify GO terms that were remarkably enriched in DEGs. The remarkably enriched GO terms in DEGs was identified with a hyergeometric test (Bonferroni-correction *P* ≤ 0.05). According to GO functional enrichment analysis, 24 terms for the up-regulated DEGs and 19 terms for down-regulated DEGs were enriched in control vs. ein control vs. S2, and 35 terms for the up-regulated DEGs and 42 terms for down-regulated DEGs were enriched in S1 vs. S2 (see Supplementary Table S3). In the category of biological processes, five terms for up-regulated DEGs (including ‘response to abiotic stimulus,’ ‘response to heat,’ ‘response to temperature stimulus,’ ‘transmembrane transport,’ and ‘carbohydrate metabolic process’) as well as two terms for down-regulated DEGs (‘protein stabilization’ and ‘electron transport’) were enriched in both control vs. S1 and control vs. S2. This result suggested that genes involved in these biological processes may play important roles responding to heat stress. In the category of molecular function, four terms for up-regulated DEGs including ‘transcription regulator activity,’ ‘chaperone binding,’ ‘signal transducer activity,’ and ‘transmembrane transporter activity’ were enriched in both control vs. S1 and control vs. S2. As for the category of cellular component, ‘external encapsulating structure’ for up-regulated DEGs as well as ‘integral to membrane’ and ‘photosystem II’ for down-regulated DEGs were enriched in both control vs. S1 and control vs. S2. This indicates that genes related to photosynthesis may be seriously affected by heat stress. Additionally, ‘ubiquitin ligase complex,’ ‘phospholipid metabolic process,’ and ‘small molecule metabolic processes’ were also enriched for up-regulated DEGs in control vs. S1. ‘Ribonucleoprotein complex,’ ‘channel activity,’ ‘alcohol metabolic process,’ and ‘protein folding’ for up-regulated DEGs were enriched in control vs. S2. Therefore, genes involved in these biological processes may also participate in responding to heat stress. Furthermore, the terms ‘response to light stimulus,’ ‘response to water deprivation’, and ‘response to salt stress’ were also enriched for up-regulated DEGs in control vs. S2, suggesting that the heat stress may have caused an efficient abiotic stress.

The significantly enriched pathways for DEGs in this study were determined by the KEGG Orthology-Based Annotation System (KOBAS) (http://kobas.cbi.pku.edu.cn/home.do). According to the KEGG pathway analysis, 7, 17, and 11 pathways were observed as significantly enriched for DEGs of control vs. S1, control vs. S2, and S1 vs. S2, respectively (see Supplementary Table S4). The pathways enriched for control vs. S1 DEGs were ‘nucleotide excision repair’ [ko03420], ‘oxidative phosphorylation’ [ko00190], ‘ubiquitin mediated proteolysis’ [ko04120], ‘photosynthesis’ [ko00195], ‘protein processing in endoplasmic reticulum’ [ko04141], ‘calcium signaling pathway’ [ko04020], and ‘phenylalanine metabolism’ [ko00360], suggesting that genes in these pathways seem to be involved in early heat sense. The top five pathways enriched for control vs. S2 DEGs were ‘carbon fixation in photosynthetic organisms’ [ko00710], ‘fatty acid metabolism’ [ko00071], ‘phenylalanine metabolism’ [ko00360], ‘plant-pathogen metabolism’ [ko04626] and ‘glycine, serine and threonine metabolism’ [ko00260]. This indicates that metabolism processes play important roles in response to heat after 5 hours. Additionally, heat shock protein genes belonging to the ‘protein processing in endoplasmic reticulum’ [ko04141] pathway was enriched in both control vs. S1 and control vs. S2, which was consistent with previous studies that plants suffering from heat stress sharply accumulate heat shock proteins to enhance heat tolerance of the plant^2^.

### Comparative analysis of genes response to heat stress in different plant species

In order to identify the similarities and differences of molecular mechanisms in response to heat stress between spinach and the other plant species, three other transcriptome profiles in response to heat stress from rice^19^, wheat^20^ and grape^21^ were compared with spinach. The comparison was carried out using publicly available datasets. In contrast to rice^19^, there were 1250 unique genes in spinach heat response transcript. In contrast to wheat^20^, there were 1340 unique genes in spinach heat response transcript. In contrast to grape^21^, there were 1701 unique genes in spinach heat response transcript. The total number of unique genes was 896 in spinach heat response transcript. Among these unique differentially expressed genes, 506 were up-regulated and 390 genes were down-regulated. GO enrichment analysis show that up regulated spinach specific genes belong to ‘response to stimulus’, ‘transmembrane transport’, ‘regulation of metabolic process’, ‘response to salt stress,’ ‘organic acid metabolic process,’ ‘channel activity’, ‘response to light stimulus’ ‘carbohydrate metabolic process’ ‘response to chemical stimulus’, ‘hydrolase activity’ and ‘regulation of transcription’. GO enrichment analysis show that down regulated spinach specific genes belong to ‘carbohydrate metabolic process’, ‘phosphorus metabolic process’, ‘metal ion binding’, ‘catalytic activity’, ‘transcription regulator activity’, ‘carotenoid metabolic process’, ‘protein kinase activity’ and ‘transferase activity’. Among these spinach unique genes, one gene related to violaxanthin was up regulated and three genes related to zeaxanthin were down-regulated during the heat treatment, which were not found in the other three species. However, it was report that xanthophylls (including violaxanthin, antheraxanthin, and zeaxanthin) could keep themostability of the membrane and lowers the harm of high temperatures^22^.

Twenty-six genes were commonly identified in all the four species. Interestingly, the expression patterns of these genes are very similar in the four plant species. Most of them were up-regulated, only four genes were down-regulated. The enriched GOs analysis indicated that these up regulated genes belong to protein folding, a common biochemical response to heat stress.

### Common molecular response of spinach under heat stress

Analysis of the transcriptome profile in plant after heat treatment indicated that the *HSP* family plays a central role in responding to heat stress^19^,^20^,^21^. HSPs families, include HSP100, HSP90, HSP70, HSP60 and small HSPs (sHSPs), are involve in folding and assembling protein, keeping protein stabilization, activating protein, and degrading protein in many normal cellular processes and under stress conditions^23^. Thirty-three DEGs were identified in our study as candidate genes for membership in different HSP families. The genes of high and middle molecular weight *HSPs* included *HSP101*, *HSP90, HSP83, HSP82*, and *HSP70*. Low molecular *HSPs* genes included *HSP18.1, HSP18.3, HSP20, HSP21.7, HSP25.3, HSP26.26*, and *HSP30*. Most of the *HSPs* were significantly up-regulated during the heat treatment, and the expression level of some were sharply increased in the 30 min heat treatment but were decreased to low levels after the 5 h heat treatment (see Supplementary Table S5). In Arabidopsis, *HSP101* homologue was involved in increasing chloroplasts thermotolerance during heat stress^24^, and *HSP 90* was significantly induced by heat, salt, or heavy metal stresses^25^. In rice, over-expression of mitochondrial *HSP70* inhibited programmed cell death (PCD) in protoplasts induced by heat- and H_2_O_2_-stress^26^. sHSPs, range from 15–42 kDa, have a conserved sequence at their C terminus, and it thought that chloroplast and mitochondrial sHSPs play a crucial role in heat tolerance^27^,^28^. In the present study, most *sHSPs* were significantly up-regulated under heat treatment, and chloroplast *HSP25.3* and *HSP26.26* increased by approximately 243-fold and 335-fold in the 30 min heat treatment. In *Chenopodium album*, chloroplast *sHSPs* are correlated with increased thermotolerance^29^. After expose to heat stress, wheat chloroplastic *sHSP* (*HSP26*) is highly expression in almost all the activated tissues and certain developmental growth stages^30^.

It is well-known that transcription factors (TFs) play an important role in biotic and abiotic stress responses (e.g., cold, high temperatures, high salinity, drought, and pathogen attacks)^2^. There were 87 DEGs including up- and down-regulated genes involved in seven TF families. These families include *HSF* (*HSF30*, *A6b*, *A5*, *A1a*, *A8*, and *B2b*), *WRKY* (*WRKY4*, *5*, *7*, *17*, *24*, *27*, *31*, *40*, *41* and *57*), *DREB* (*DREB 2A* and *2B*), basic leucine zipper (*bZIP9*, *11*, *12*, and *37*), *NAC* (*NAC1* and *68*), *MYB* family (*MYB 1*, *108*, *306*, and *811*), and *C2H2* (Table 3).

*HSFs* have been shown to be involved in basal and acquired thermotolerance^31^. According to sequence homology and domain architecture, plants HSF proteins are classed into are three conserved categories: A, B, and C^32^. In the present study, sixteen DEG were similar to *HSFs*, including *HSF30*, *HSFA6b*, *HSFA5*, *HSFA1a*, *HSFA8*, and *HSFB2b*, and fourteen of these genes were induced during the heat treatment. It was reported that *HSFA1a* and *HSF30* could govern the expression of some *HSPs*^31^,^33^,^34^,^35^. In Arabidopsis, *HsfA4a* and *HsfA8* function as sensing elements of the reactive oxygen species (ROS) during the heat stress response^36^,^37^. More and more evidences indicate that several *WRKY* genes are involved in the regulation of various abiotic stress responses, including heat^38^. In Arabidopsis, *WRKY7, 25* were regulated by heat shock^39^,^40^. In this study, thirteen DEGs similar to *WRKY 7*, *17*, *27*, *31, 40*, and *57* were up-regulated in S1 and twelve DEGs similar to *WRKY 7*, *24*, *31*, and 41 were up-regulated in S2 compared to control sample. The dehydration-responsive element binding protein (DREB) family plays an important role in the responses to abiotic stress in plants^41^,^42^. In Arabidopsis, *DREB2A* and *DREB2B* are induced transiently within 1 h after heat stress, but *DREB2B* expression continued for 12 h after heat treatment^41^. It was reported that *OsDREB2B* improve the thermotolerance in rice^42^. In this study, *DREB2A* was up-regulated in S1 and *DREB2B* was up-regulated in S2. *Basic leucine zipper* (*bZIP*) transcription factors, plant-specific *NAC* family transcription factors, *MYB* transcription factors and *C2H2-type* zinc finger transcription factors have been implicated in various biological functions, including biotic and abiotic stress^43^,^44^,^45^,^46^. In the present study, twelve *bZIP* , nine *NAC* domain, sixteen *MYB* and eleven *C2H2* genes were heat-regulated in spinach leaves, and the expression patterns were show in Table 3. These results should be helpful for explaining the important function of the *bZIP*, *NAC*, and *C2H2* family genes in the heat response of spinach leaves.

When suffering from heat stress, plants could activate various stress-responsive signal transduction pathways to generate a series of innate defensive reactions^47^. Calcium signal is important in response to heat stress, and heat stress causes transient rise of Ca^2+^ levels in many plants^48^. In Arabidopsis, the calmodulin *AtCaM3* is a required signaling element in heat stress^49^ and may activate different transcription factors, such as WRKY39^50^ and HSFs^51^. In wheat, a number of genes related to calcium signal pathways were heat regulated, including annexin, calcium-binding proteins (CBPs), calcium-dependent protein kinases (CDPKs), voltage-gated calcium channel activity, Ca^2+^ -binding protein EF hand, CBL (calcineurin B-like protein), and CIPK (CBL-interacting protein kinase)^20^. In this study, twenty DEGs were highly homolugous to calcium signaling genes including CaM, CDPK, CBK, and CBL, and sixteen of these genes were up-regulated in control vs.S1. Mitogen-activated protein kinase (MAPK) cascades are thought to act as useful role in many responses to abiotic stress^47^. The activation of HAMK in tobacco cells was observed together with HSP70 accumulation, suggesting that HAMK may induce a heat signaling cascade via *HSP* genes^52^. It was shown that the transcript abundance of the tomato *SlMAPK* increases after heat treatment^53^. In this study, fourteen DEGs were highly similar to *MAPK* genes, including *MAPKKK2*, *MAPK2*, *MAPK6*, and *MPAK18*. All the genes encoding *MAPKs* were up-regulated during the heat treatment compared with control samples, except those encoding *MAPKKK2*.

The differential expression analysis revealed that the genes related to secondary metabolism exhibited complex and significant expression changes under heat stress^2^. Phenolics, such as flavonoids, anthocyanins, lignins, play a variety of roles in response to abiotic stresses^2^. In rice, genes for *PAL* and *4-coumarate CoA ligase* (*4CL*) were induced by heat stress^54^. In this study, seventeen DEGs were found to be involved with phenolics. Among them, one gene for *PAL* and three genes for *4CL* were up-regulated during the heat treatment, two genes for caffeic acid *3-O-methyltransferase* and two genes for *dihydroflavonol 4-reductase* were induced after the 5 h heat treatment, and the other genes were repressed. Genes involved in terpenoid metabolism have been investigated in response to various stresses and defense signals^55^. In this study, six DEGs were related to terpenoid biosynthesis after heat treatment. The genes encoding 3*-hydroxy-3-methy lglutaryl-coenzyme A reductase, protein-S-isoprenylcysteine O-methyltransferase B*, and *geranylgeranyl hydrogenase* were up-regulated after the 5 h heat treatment, and the other DEGs were repressed.

It was found that heat stress could induce some resistant protein involved with other abiotic or biotic stress^2^. Late embryogenesis abundant (LEA) proteins can protect the citrate synthase from dehydrating conditions, such as heat and drought stress^56^. In sugarcane leaves, three low-molecular-weight dehydrin proteins were induced in response to heat stress^57^. In this study, three DEGs for a NBS-LRR type resistance protein, disease resistance protein involved in disease stress, were up-regulated during the heat treatment. Seven DEGs for sugar transporter, LEA protein, salt tolerance-like protein, and water channel protein involved in osmotic stress were up-regulated after 5 h heat treatment. Oxidative stress is a kind of secondary stress during the heat stress response, which produces a large number of ROS^2^. ROS concentrations are regulated by a series of antioxidant enzymes, such as superoxide dismutases (SOD), ascorbate peroxidase (APX), dehydroascorbate reductase (DHAR), and catalase (CAT)^38^. In this study, eight DEGs related to oxidative stress were identified. Six DEGs encoding Cu/Zn superoxide dismutase (Cu/Zn-SOD), Fe superoxide dismutase (Fe-SOD), dehydroascorbate reductase (DHAR), and catalase (CAT) were found to be up-regulated and two DEGs for peroxidase (POD) were significantly repressed during the heat stress. It was reported that CAT primarily acts as a scavenger of ROS in plants under heat stress^58^. In grape, *DHAR* was up-regulated by 3.18-fold^30^. Therefore, these genes may play an important role in the heat stress response of spinach leaves.

## Conclusion

Spinach varieties ‘Island’, come from Seminis Vegetable Seeds Inc, are suitable for summer periods. The spinach leaf de novo transcriptome under heat stress was analyzed using NGS-based RNA-Seq technology. Differentially expressed genes (DEGs) were determined with FPKM method. The DEG for controls vs S1 was 986, the DEG for controls vs S2 was 1741 and the DEG for S1 vs S2 was 1587. Compared with control, there were 550 and 1131 up-regulated unigenes in the S1 and S2 samples, respectively. In these identified heat-responsive genes, calcium signaling molecule and some transcription factors play an important role in response to early heat stress. Comparative transcriptome analysis found a number of unique genes in spinach heat response transcript. Among these unique differentially expressed genes, we speculate some genes involved in salt stress, organic acid metabolic and carotenoid metabolic deserve to pay more attention, because they represent special mechanism in spinach response to heat stress. In a word, these results were helpful for understanding the molecular adaptation mechanism of spinach during the heat stress.

## Methods

### Plant materials and heat treatment

*S. oleracea* L. var. Island was selected in this study for its high heat tolerance. The seeds were surface-sterilized and sown into soil in plastic pots, and then the seedlings were cultured in a growth chamber where they were maintained at 25 °C (14 h/10 h day/night cycle, relative humidity ranging from 50%–70%, and light intensity at 300 μmol m^−2^ s^−1^). Seedlings with four true leaves were then cultured in the same chamber with the same conditions except with the temperature at 35 °C. After 0 (control), 30 min (S1), and 5 h (S2) of heat treatment, the third true leaves were collected from all three different samples. The samples were immersed in liquid nitrogen and stored at −80 °C for RNA extraction and quantitative real-time PCR analysis.

### RNA extraction, cDNA library construction, and Illumina sequencing

Total RNA from different samples was extracted using the TRIZOL reagent (Takara, China). Agilent 2100 Bioanalyzer was used to test the intergrity of RNA, which revealed that all RNA samples were integrate (integrity number value >8.0). Three libraries (control, S1 and S2) were prepared according to the manufacturer’s instructions of the Truseq^TM^ RNA sample prep Kit (Illumina, Inc. San Diego, CA, USA). Firstly, mRNA was enriched using magnetic beads containing poly-T molecules. Subsequently, the enriched mRNA was broken into short fragments and then reverse transcribed into cDNA using PrimeScript 1^st^ Strand cDNA Synthesis Kit (TaKaRa, China). Finally these cDNA fragments were carried out end repair and were ligated with Illumina adapters. Three libraries with an insert size of 200 bp were constructed and then sequenced using the Illumina HiSeq^TM^ 2500.

### Short reads de novo assembly

Firstly, the raw reads were cleaned by removing adaptor sequences, empty reads, and low quality (phred quality below 5). Then, transcriptome de novo assembly was carried out using Trinity (http://trinityrnaseq.sourceforge.net)^59^. The clean reads were first assembled to produce longer fragments named as contigs. In order to avoid obtain contigs from the same transcript, the paired-end reads were used to calculated the distance and relation among these contigs. Finally, contigs were connected until it cannot be extended on either end, and then these sequences need to perform a redundancy removal process with sequence clustering software to obtain non-redundant transcripts. These non-redundant transcripts were named as unigenes. Bowtie was used to map the clean reads back to the assembled transcripts.

### Gene function annotations and classifications

The NCBI non-redundant (nr), Swiss-Prot, Cluster of Orthologous Groups (COG), and Kyoto Encyclopedia of Genes and Genomes (KEGG) databases were used to annotated all the unigenes with local BLAST programs (E value <1.0E-5). For the nr annotation results, the Blast2GO program was applied to classify unigenes based on Gene Ontology (GO) terms, and then the WEGO tool was used to draw the GO tree. The COG database was also used to predict the functional classification of the unigenes. All unigenes were match with the KEGG pathway database to predict pathway unigenes participated with BLASTx (E value <1.0E-5).

### Identification of differentially expressed genes (DEGs)

Differentially expressed genes (DEGs) of different libraries were analyzed using the FPKM (the fragments per kilobase of exon region in a given gene per million mapped fragments) method and DEGs were identified with edgeR package^60^. Briefly, FPKM was used to determine the number of mapping reads for every unigene, and also to assess the unigene expression levels. The trimmed mean of M-values (TMM) method was used to calculate normalization factors. The negative binomial distribution methods were applied to calculate p values. The Benjamini-Hochberg methods were chosen to adjust for multiple tests. Unigenes were determined to be significantly differentially expressed if they had a p-value <0.05 and a false discovery rate (FDR) <0.001. Besides, GO functional enrichment and KEGG pathway enrichment analysis of DEGs were carried out via comparison with the whole-transcriptome background using the formula described in previous studies (Bonferroni-corrected p-value ≤ 0.05)^18^.

### Validation by real-time quantitative RT-PCR

Thirteen DEGs were chosen to confirm that they involve in responding heat stress using RT-qPCR method. Primer Premier 3.0 software was used to design gene-specific primers on the basis of the selected unigene sequences (see Supplementary Table S6). RT-qPCR was carried out on an ABI System using Fast Start Universal SYBR Green Master Mix (Roche). PCR amplifications included the following conditions: 95 °C for 3 min, followed by 40 cycles of 95 °C for 15 s, and then 60 °C for 30 s. A melting curve was obtained from 60 to 95 °C by increasing the temperature stepwise by 0.5 °C every 5 s. This was done to test the specificity of the amplified product. The expression levels of selected DEGs were normalized by comparing with an internal reference gene, 18SrRNA. Their relative expression level was calculated via the 2^−ΔΔCt^ method^61^. All RT-qPCR were repeated in three biological and three technical replications.

## Additional Information

**How to cite this article**: Yan, J. *et al*. De novo transcriptome sequencing and gene expression profiling of spinach (Spinacia oleracea L.) leaves under heat stress. *Sci. Rep*. **6**, 19473; doi: 10.1038/srep19473 (2016).

## Supplementary Material

Supplementary Table S1

Supplementary Table S2

Supplementary Table S3

Supplementary Table S4

Supplementary Table S5

Supplementary Table S6

## Figures and Tables

**Table 1:** Total number of original and cleaned reads that were mapped to their respective assembled transcripts.

Map to Genome	S1 sample		Control sample		S2 sample	
	Number of reads	percentage	Number of reads	percentage	Number of reads	percentage
Total reads	44682577	100.00%	42290681	100.00%	46227640	100.00%
Total base pairs	4349881212	100.00%	4122064627	100.00%	4508391358	100.00%
perfect match	30160739	67.50%	28842244	68.20%	31120447	67.32%
< = 2bp mismatch	5478084	12.26%	4931093	11.66%	5246837	11.35%
unique match	18020483	40.33%	17592923	41.60%	18407846	39.82%
multi-position match	19770071	42.35%	17267285	40.83%	20099778	43.48%
Total unmapped reads	7694340	17.22%	6884923	16.28%	8390317	18.15%
Total mapped reads	36988237	82.78%	35405758	83.72%	37837323	81.85%

**Table 2:** The length distribution of spinach, rice and Arabidopsis.

	0–400 bp	401–1000 bp	1001–3200 bp	>3200 bp
Spinach	5841 (9.83%)	26684 (44.91%)	23655 (39.81%)	3233 (5.44%)
Rice	6774 (10.21%)	15277 (23.03%)	36950 (55.7%)	7337 (11.06%)
Arabidopsis	3465 (8.32%)	10137 (24.33%)	25065 (60.15%)	3004 (7.2%)

**Table 3:** The number of DEG identified as transcription factors in spinach leaves.

Category	Total	S1^a^		S2^b^	
		Up-regulated	Down-regulated	Up-regulated	Down-regulated
*HSF*	16	12	1	6	1
*WRKY*	15	13	2	12	0
*DREB*	8	3	0	6	0
*bZIP*	12	9	0	9	3
*NAC*	9	5	3	4	1
*MYB*	16	7	9	3	4
*C2H2*	11	9	3	9	0

^a^Spianch exposed to 35 °C for 30 min.

^b^Spinach exposed to 35 °C for 5 h.
